# Adaptor protein Shc acts as an immune-regulator for the LPS-stimulated maturation of bone marrow-derived dendritic cells

**DOI:** 10.1186/1471-2172-12-32

**Published:** 2011-05-25

**Authors:** Kuang-Den Chen, Li-Wen Hsu, Shigeru Goto, Chin-Wei Yeh, Toshiaki Nakano, Chia-Yun Lai, Yen-Chen Chang, Chiung-Hui Hou, Chih-Chi Wang, Yu-Fan Cheng, King-Wah Chiu, Chih-Che Lin, Chao-Long Chen

**Affiliations:** 1 Center for Translational Research in Biomedical Sciences, Liver Transplantation Program, Departments of Surgery, Kaohsiung Chang Gung Memorial Hospital and Chang Gung University College of Medicine, Kaohsiung, Taiwan; 2 Department of Chemistry, National Cheng Kung University, Tainan, Taiwan; 3 Iwao Hospital, Yufuin, Japan; 4 Graduate Institute of Clinical Medical Sciences, Chang Gung University College of Medicine, Kaohsiung, Taiwan; 5 Diagnostic Radiology, Chang Gung Memorial Hospital-Kaohsiung Medical Center, Kaohsiung, Taiwan; 6 Hepato-gastroenterology, Chang Gung Memorial Hospital-Kaohsiung Medical Center, Kaohsiung, Taiwan

## Abstract

**Background:**

The Shc isoforms is known to mediate immune responses and has been indicated as a negative regulator of autoimmunity and lymphocyte activation. We aimed to evaluate the immune-regulatory role of Shc in rat bone marrow-derived DCs in the maturation process triggered by LPS.

**Results:**

We found that, in response to LPS, expression of Shc proteins was induced and that neutralization of Shc inhibited the LPS-induced transient phosphorylation of p52Shc on pTyr239/240 in DCs of Lewis (LEW; RT1^l^) rats. Moreover, the significantly enhanced expression of IL-10 and the surface level of costimulatory molecule CD80, as well as suppressed expression of IL-6 and IL-12 in the Shc-silenced DCs were also observed. Similar IκB phosphorylation occurred in Shc-silenced DCs primed by LPS, indicating Shc is not associated with NF-κB pathway. We further demonstrate that Shc blockade on LPS-treated DCs results in significant increase of the overall STAT3 phosphorylation and the relative levels of phospho-STAT3 in the nuclear fraction. STAT3 activation by LPS with or without Shc blockade was totally abolished by SU6656, a selective Src family kinases inhibitor, underscoring the critical role of Src-mediated activation.

**Conclusions:**

We conclude that Shc blockade in LPS-primed DC leads to the development of tolerogenic DC via Src-dependent STAT3 activation and that adaptor protein Shc might play a pivotal role in mediating immunogenic and tolerogenic properties of DCs.

## Background

Dendritic cells (DCs) are the most important APC that play a crucial role in bridging innate resistance and adaptive immunity [1,2]. Immature DCs reside in peripheral tissues, where they release soluble mediators (cytokines, chemokines and IFNs) that participate in innate inflammatory responses during infection [2,3]. Upon capturing antigens, DCs migrate to the lymph nodes and present processed antigenic epitopes to T cells, resulting in their activation and further expansion [4,5]. A variety of signals induce DC maturation. Mature DCs express high levels of antigen presenting and co-stimulatory molecules and certain cytokines critical for the nature of the T cell response. For instance, Th1-type T cell responses need inflammatory IL-12 produced by DCs. Conversely, DCs can also produce anti-inflammatory cytokines, such as IL-10 [6-8], which influences the DC maturation process by down-regulating IL-12 production and thus interfering with the Th1-type T cell responses [4,9-11]. Moreover, IL-10-producing DCs also promote immune tolerance by modulating the suppressive effects of regulatory T cells [12,13]. Accordingly, there has been considerable interest in influencing the DC maturation process to direct T cell responses to a desired type (i.e., Th1 vs. Th2/3) for translational purposes.

A useful system for the study of DCs in culture is the use of monocyte-derived DCs obtained *in vitro *by GM-CSF and rIL-4 treatment of peripheral CD14^+ ^monocytes. These cells can produce high amounts of cytokines such as IL-6 and IL-12 when stimulated with LPS [14]. Some evidence suggests an involvement of Src-family tyrosine kinases (SFKs) in the signaling pathway triggered by LPS. In monocytes, LPS activates the Src-family kinase Lyn associated with CD14, a glycosyl phosphatidylinositol (GPI)-anchored molecule that cooperates with toll-like receptor 4 (TLR4) in LPS binding on the surface of these cells [15]. However, the complexity in the engagement of TLRs by LPS leading to interactions with intracellular adaptor proteins and their associated kinases is still under investigation.

Shc adaptor proteins are substrates of receptor tyrosine kinases, and signal events initiated by their phosphorylation culminate in Erk and Jnk activation [16,17]. Among the three related Shc genes, ShcA is ubiquitously expressed, whereas ShcB and ShcC are restricted to cells of neural origin, and we describe ShcA here as Shc based on this tissue restriction. Shc is expressed as three isoforms of 46, 52 and 66 kDa derived from ShcA via post-transcriptional splicing, which display the PTB-CH1-SH2 Shc family signature, with an added N-terminal CH2 domain in p66ShcA and a truncated PTB domain in p46ShcA. The PTB and SH2 domains both bind tyrosine-phosphorylated peptides and associate with activated receptor kinases [18]. Recently, it has been found that the defects of pp66ShcA in T cells of p66ShcA^-/- ^mice display enhanced proliferative responses to T-cell antigen receptor (TCR) agonists, suggesting a potential role of p66ShcA in lymphocyte homeostasis [19]. The p66ShcA^-/- ^mice also develop a lupus-like autoimmune disease, which implies a possible key role of p66ShcA in regulating immunologic tolerance and the development of systemic autoimmunity. However, there has been no definite evidence of a role for Shc proteins in DC maturation, cytokine production or the expression of co-stimulatory molecules triggered by LPS.

In the present study, we briefly address the role of Shc proteins in the maturation process triggered by LPS in rat bone marrow-derived DCs, and we evaluate their contribution in the context of LPS-induced TLR4 signaling.

## Results

### Transient activation and sustained induction of Shc proteins

**Figure 1:**
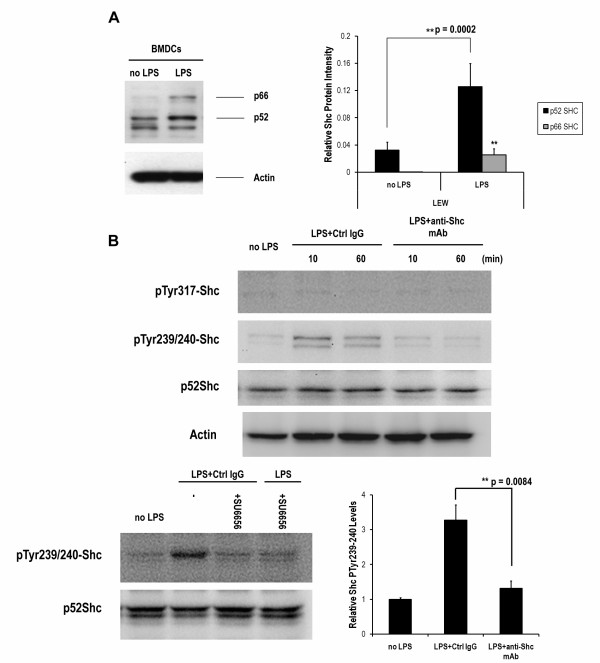
**LPS stimulates Shc protein accumulation and activation in bone marrow-derived DCs**. Purified DCs were stimulated with LPS (0.5 μg/ml), and Shc protein accumulation was measured at 48 h (A). For Shc activation, DCs were treated with LPS (0.5 μg/ml) with mouse control IgG or anti-Shc mAb (2 μg/ml) for 10 or 30 min, or with LPS (0.5 μg/ml) in the presence of 10 nM SU6656 and/or mouse control IgG (2 μg/ml) for 10 min, as indicated (B). Cell lysate (30 μg) from each sample was subjected to Western blot with anti-Shc, anti-phosphotyrosine 317-Shc (pTyr317-Shc) and anti-phosphotyrosine 239/240-Shc (pTyr239/240-Shc) mAb. The bound antibodies were detected by the ECL system. The significant change of pTyr239/240-Shc was observed and the statistics at 10-min was presented. The results are representative of three independent experiments. (n = 3; **, P < 0.01)

To test whether LPS can activate Shc, bone marrow-derived DCs were treated with 0.5 μg/ml LPS for 48 hrs. When dendritic cells obtained from LEW rats were cultured with rGM-CSF and rIL-4, p46Shc and p52Shc were expressed in immature DCs, but the inducible form of p66Shc was not observed, as shown by Western blot analysis (Figure 1A). At 48 h after the addition of endotoxin, the expression of p66Shc was induced, and the accumulation of p52Shc was also increased. However, no significant change was observed in p46Shc accumulation.

Shc phosphorylation mechanisms were also examined in the LPS-induced maturation of DCs. Figure 1B shows that p52Shc protein was transiently phosphorylated on tyrosine residues 239/240 but not 317. We further evaluated whether the addition of anti-Shc mAb could block the phosphorylation events of Shc in DCs exposed to LPS. As shown in Figure 1B, the LPS-induced transient phosphorylation of Shc protein in YY239/240 was down-regulated by 24-h preincubation of anti-Shc monoclonal antibody. This result suggests that the anti-Shc mAb we used could act as a blocking antibody for Shc neutralization. Moreover, we evaluated the role of non-receptor Src-family kinases (SFKs) in Shc phosphorylation by using a small molecule Src-family kinase inhibitor SU6656 while DCs was exposed to LPS. The inhibition of Shc phosphorylation by SU6656 indicating the involvement of SFKs activity in LPS-triggered Shc signaling events. Therefore, tyrosine phosphorylation on positions 239/240 of Shc is Src-dependent.

### Effect of Shc silencing on the response of DCs to LPS exposure

**Figure 2:**
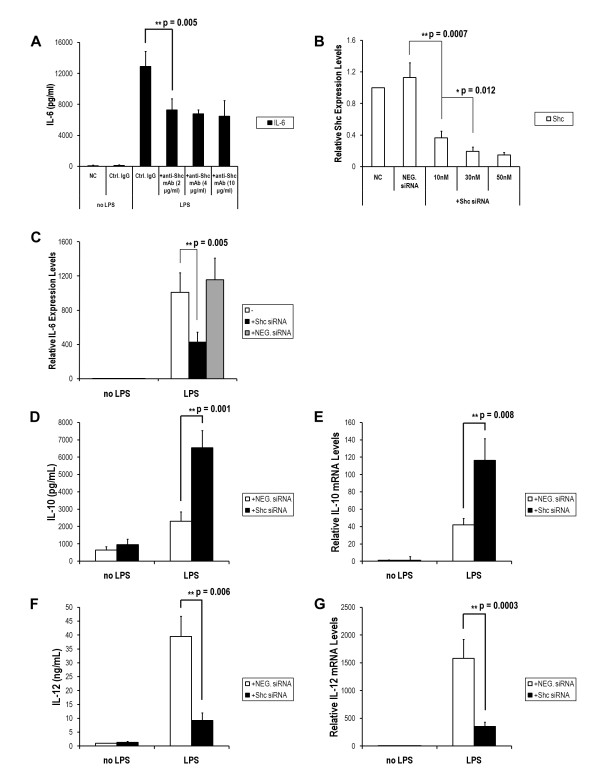
**Effects of Shc blockade on the secretion and mRNA expression of IL-6, IL-10 and IL-12 in DC maturation**. Purified DCs were cultured with or without anti-mouse Shc mAb at various concentrations, as indicated, and stimulated with LPS (0.5 μg/ml) for 24 h and the IL-6 secretion was measured in triplicate supernatants by ELISA (A). DCs were transfected with Shc siRNA or negative control siRNA (NEG. siRNA) as described in the Materials and Methods. Quantitation of the RNAi efficiency for Shc silencing was analyzed by quantitative PCR to confirm the blockade effect of the Shc siRNA 24 h after transfection (B). The relative expression levels of IL-6 mRNA were measured in BMDCs stimulated by LPS in the absence or presence of Shc siRNA (50 nM), or NEG. siRNA (50 nM) for 24 h. Cells were collected, RNA was isolated, and IL-6 mRNA was determined by RT-PCR (C). Moreover, the amount of IL-10 and IL-12 were also determined by ELISA and RT-PCR. The data show enhanced secretion and mRNA level of IL-10 (D, E) and reduced production of IL-12 (F, G) from DCs in the presence of Shc siRNA. Each column represents the mean ± SE of the three independent experiments (n = 3; *, P < 0.05; **, P < 0.01).

To evaluate the role of Shc proteins in DC cytokine production, a neutralizing anti-Shc mAb was added to immature DCs, which concomitantly treated with LPS for 24 h. As shown in Figure 2, incubation of DCs with anti-Shc mAb but not with control mouse IgG in the presence of LPS for 24 h elicited a lower level of IL-6 secretion (Figure 2A). To determine whether the endogenous Shc directly involved in LPS-induced IL-6 production, the Shc siRNA was used. Shc siRNA dose-dependently inhibited the expression of Shc mRNA expression (Figure 2B). Figure 2C also shows that LPS-induced IL-6 production was inhibited by Shc siRNA. Decreased secretion of IL-6 by 2.5-fold in DCs stimulated with LPS and concomitantly with transfected Shc siRNA (Figure 2C).

In addition to IL-6, LPS induces DC production of various cytokines, including IL-10 and IL-12. Therefore, the homeostasis of inflammation versus immune suppression controlled by DCs via the balance of produced cytokines would be crucial, so we determined the role of Shc in IL-10 and IL-12 secretion. DCs were treated with LPS (0.5 μg/ml) for 24 h in the presence of transfected Shc siRNA (50 nM) or NEG. siRNA (50 nM), and the secretion of IL-10 and IL-12p70 in the supernatant were measured by ELISA. The mRNA expression of IL-10 and IL-12p40 was also determined by quantitative RT-PCR. We found that LPS induced substantial secretion of IL-10 and increased expression of IL-10 mRNA by DCs. Notably, the LPS-induced production of IL-10 was significantly enhanced in the presence of Shc siRNA (Figure 2D and 2E). In contrast, cultured DCs showed vigorous secretion of IL-12p70 and mRNA expression of IL-12p40 in response to LPS treatment, while the IL-12 levels were significantly suppressed by Shc siRNA (Figure 2F and 2G). Therefore, Shc-mediated signaling appears to be crucial for regulation of IL-10 and IL-12 production by DCs upon LPS stimulation.

### Enhanced expression of T cell co-stimulatory molecule CD80 on Shc-silenced DCs in response to LPS exposure

**Figure 3:**
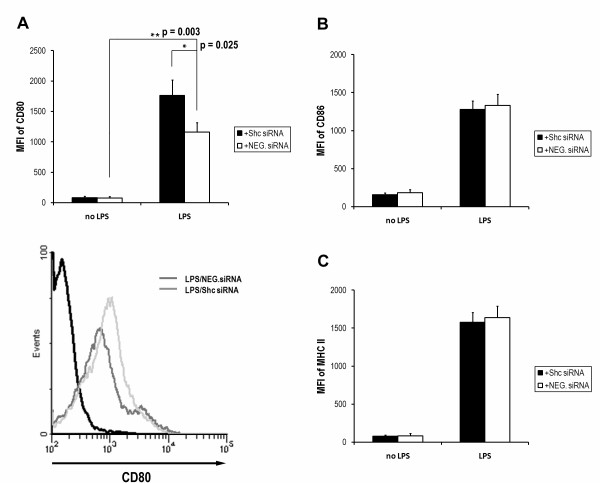
**Surface marker measurements of DC phenotype for the effects of Shc blockade on LPS-primed maturation**. DCs cultured in medium only were immature, with relative low expression of MHC-II and co-stimulatory markers CD80 and CD86 (no LPS). The administration of LPS induced similar levels of DC maturation, indicated by up-regulation of MHC-II, and CD86, in the presence of transfected Shc siRNA (black) or NEG. siRNA (white) (B and C). However, the co-inhibitory marker CD80 was significantly enhanced when Shc was silenced in LPS-treated DCs (compared to NEG. siRNA) (A). The lower panel of 3A shows histogram overlay of CD80 expression in control (black), LPS-exposed with NEG. siRNA (gray) and LPS-exposed with Shc siRNA (light gray) transfection measured by flow cytometry. Each column represents the mean fluorescence intensity (MFI) ± SE of the three independent experiments (n = 3; **, P < 0.01; *, P < 0.05).

DCs undergo a maturation process while the host detects a microbial infection, which enhances their ability to process and present exogenous antigen. Optimal antigen presentation of DCs requires the expression of specific membrane protein markers. Maturation of DCs is associated with profound changes in the surface phenotype of protein markers, including an up-regulated expression of co-stimulatory and MHC class II molecules [20,21]. During maturation, the co-stimulatory molecules (CD80, CD86) are required for T cell priming and produce cytokines that direct T cell differentiation, whereas processed antigen fragments are present to the surface using MHC-II molecules. In order to evaluate the effects of Shc blockade on co-stimulatory and MHC class II molecules during DC maturation, the immature DCs were transfected with Shc siRNA prior to LPS treatment and the surface protein expression was measured by flow cytometry. CD80, CD86 and MHC-II protein levels were increased significantly in DCs expose to LPS for 24 h. Moreover, we found that DCs treated with LPS for 24 h with transfected Shc siRNA display enhanced level of CD80 (P < 0.05), but not CD86 or MHC-II (Figure 3), indicating that CD80 was selectively enhanced during LPS-triggered maturation with Shc blockade.

### LPS-induced STAT3 phosphorylation and nuclear translocation are highly enhanced by Shc silencing in DCs

**Figure 4:**
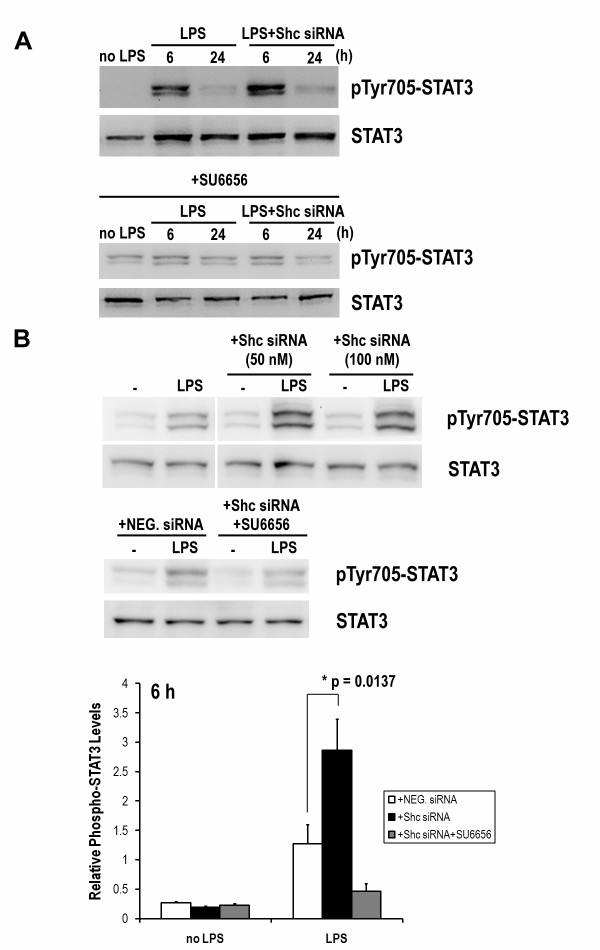
**STAT3 phosphorylation activated by LPS is significantly enhanced by Shc blockade**. DCs from Lewis rats were stimulated with LPS (0.5 μg/ml) in the presence of Shc siRNA (50 nM, 100 nM) or NEG. siRNA. Cells were harvested and Western blot analysis was performed for phospho-STAT3 at Tyr-705 and for total STAT3. Additional treatment of 10 nM SU6656 indicated total inhibition of STAT3 phosphorylation at Tyr-705. Each column represents the mean ± SE of the three independent experiments (n = 3; *, P < 0.05).

**Figure 5:**
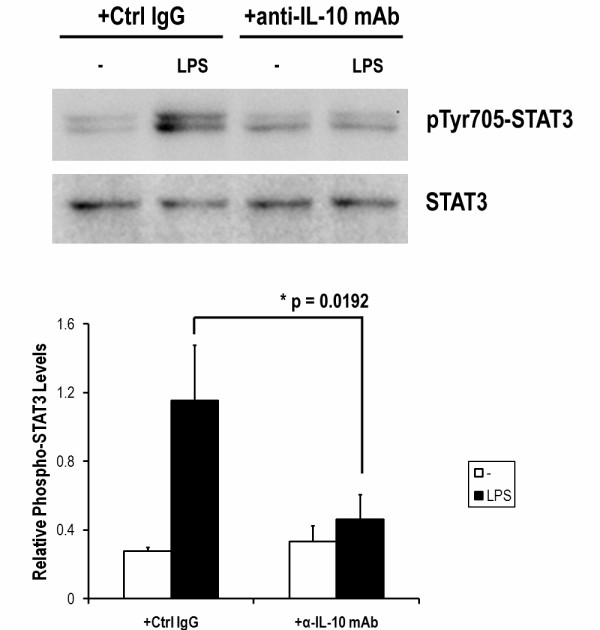
**LPS-induced STAT3 phosphorylation was inhibited by IL-10 neutralizing mAb on DCs**. DCs from Lewis rats were stimulated with LPS (0.5 μg/ml) in the presence of control IgG (Ctrl IgG, 2 μg/ml), anti-IL-10 mAb (2 μg/ml) or anti-IL-10 mAb combined with Shc silencing for 6 h. Cells were harvested and Western blot analysis was performed for phospho-STAT3 at Tyr-705 and for total STAT3. Anti-IL-10 mAb significantly inhibited the LPS-induced STAT3 phosphorylation in both DCs and Shc-silenced DCs indicated the dependence of secreted IL-10 in STAT3 activation. Each column represents the mean ± SE of the three independent experiments (n = 3; *, P < 0.05).

We further investigated STAT3 phosphorylation in LPS-induced DCs with or without Shc blockade. As shown in Figure 4, STAT3 was highly phosphorylated at 6 h in LPS-stimulated DCs, and its phosphorylation was almost down to basal after 24 h. The phosphorylation level of STAT3 was significantly enhanced in DCs transfected with Shc siRNA but not NEG. siRNA. Moreover, the involvement of LPS-induced IL-10 secretion was also examined by using IL-10 neutralizing antibody (Figure 5). Our data suggests the LPS-induced STAT3 activation and the enhanced STAT3 activation via Shc blockade are IL-10 dependent. Furthermore, SU6656 totally abolished the induced STAT3 phosphorylation indicating the involvement of SFKs activity in STAT3 activation of LPS-stimulated DCs.

**Figure 6:**
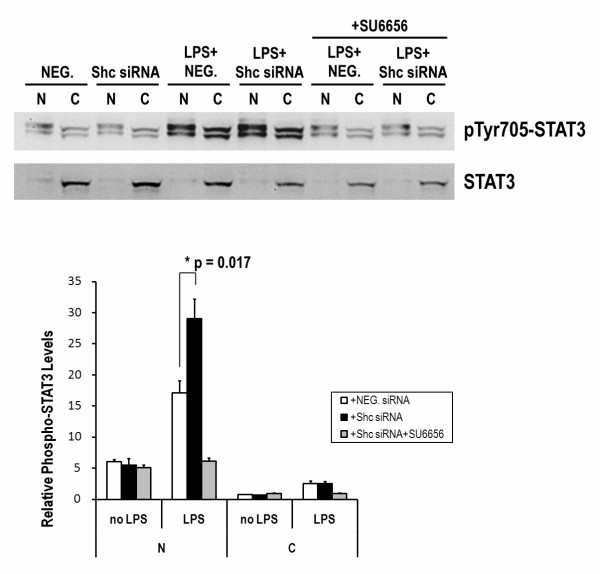
**Cell fractionation analysis shows significant increased level of phospho-STAT3 in the nucleus (N) but not cytoplasm (C) on LPS-treated DCs with Shc silencing**. DCs from Lewis rats were stimulated with LPS (0.5 μg/ml) in the presence of Shc siRNA (50 nM) or equal amount of NEG. siRNA. Nuclei were pelleted and the supernatant was collected as described in Materials and Methods. The cytoplasmic fraction (C) and the nuclear fraction (N) were analyzed by SDS/PAGE and Western blot for the presence of endogenous STAT3. The phosphorylation levels of endogenous STAT3 at Tyr-705 were significantly increased in both N and C fractions after LPS treatment for 6 h. However, the significant enhancement of STAT3 phosphorylation was observed only in the N fraction LPS-treated DCs in the presence of Shc siRNA. Each column represents the mean ± SE of the three independent experiments (n = 3; *, P < 0.05).

The nuclear translocation of phospho-STAT3 was measured. As shown in Figure 6, Shc blockade promotes nuclear localization of phospho-STAT3. STAT3 was detected mainly in the cytosolic fraction of DCs with a variety of treatment conditions. The relative phosphorylation levels of STAT3 in both cytosolic and nuclear fractions were increased by LPS treatment for 6 h. Moreover, the relative phosphorylation level of STAT3 in the nuclear fraction (N) but not in the cytosol (C) was highly enhanced by LPS treatment with Shc blockade. This result indicates that more STAT3 phosphorylated on Tyr-705 is generated into the nucleus in LPS-induced DCs with Shc blockade. The activation of STAT3 by LPS with or without Shc blockade was totally abolished by SU6656 both in N and C fractions.

## Discussion

We show that expression of Shc proteins was induced by LPS and that Shc blockade inhibited LPS-primed phosphorylation of p52Shc on tyrosine residues. IL-6 and IL-12 production triggered by LPS were suppressed by Shc blockade. Moreover, the expressions of costimulatory molecule CD80 and of IL-10 were enhanced while silencing endogenous Shc on LPS-induced DCs. However, the LPS-induced IL-10 production would be totally abolished in the presence of SU6656, implying that the signals transducing via adaptor protein Shc negatively regulate the IL-10 expression and underscoring the complexity of the role of SFKs.

Adaptive immunity evolved from an ancient innate defense mechanism common to most microorganisms. Therefore, it is not surprising that sensing and signal transduction in innate immunity is the starting point of the specific immune response. APCs, such as macrophages/monocytes and DCs, activated by various antigens of bacteria [22,23] and other microorganisms [24-26] through Toll-like receptors (TLRs), could be central to this process that integrates innate information and conveys it to lymphocytes [27]. Adaptor proteins differ from other signaling molecules in their lack of inherent catalytic activity, but they are primarily composed of modular domains that bind and act as scaffolds for the organization of macromolecular complexes, and they recruit other signaling proteins for correct localization. They play important roles in the integration and propagation of signals that lead to lymphocyte development and homeostasis [28]. Therefore, the mechanisms underlying TLR-mediated activation of APCs have been extensively analyzed, but the downstream signaling pathways governing the release of inflammatory mediators in response to LPS are yet to be understood.

The LPS-stimulated secretion of large amounts of pro-inflammatory cytokines requires the recruitment of intracellular adaptor molecules. For example, myeloid differentiation primary response gene 88 (MyD88) is recruited to activate the NF-κB and MAPK pathways via TNF receptor-associated factor 6 (TRAF6) and the IL-1 receptor-associated kinases (IRAKs) [29], and a second pair of Toll-IL-1-resistance (TIR) domain-containing adaptors inducing interferon-beta (TRIF) and TRIF-related adaptor molecule (TRAM) induce signaling via TRAF3 and the interferon regulatory factors (IRFs) to activate the interferon response [30]. However, there is growing evidence that other adaptor molecules could also make important contributions to the inflammatory responses. The role of p52Shc in transducing signals for proliferative responses to TCR agonists has been reported [31] and the negative regulation in T-cell and B-cell activation via p66Shc using p66^-/- ^mice has also been illustrated [19].

The involvement of Shc members in signaling pathways have been well characterized [17,32,33]. The CH1 domain has three tyrosine residues, 239/240 and 317 in p52Shc, which upon phosphorylation interact with Grb2 and have been proposed to be differentially coupled to Fos-dependent proliferation signaling (Y317) and to Myc-dependent survival pathways (YY239/240) in B-cell [32] and T-cell activation [34]. In the present study, we provide new insight into the role of Shc in the maturation process triggered by LPS in DC, and we evaluate their contribution in the context of LPS-induced TLR4 signaling. The proximal signaling events occurring through Shc in association with LPS-induced maturation of DC were first defined. Our study was based on the primary observation in accumulation of Shc proteins after a 48-h LPS treatment. Strong suppression of IL-6 and IL-12 production by Shc siRNA as well as anti-Shc mAb in DC suggests the role of Shc signaling in promoting the pro-inflammatory properties of LPS-stimulated DC. However, the Shc-silenced DC stimulated with LPS also exhibited increased levels of costimulatory molecules and MHC II antigen stimulation. This indicates that the tolerogenic state of LPS-primed DC under Shc silencing could be determined by unravel factors through IL-10, a cytokine which has also been found in contributing to hCG-induced tolerogenic phenotype of DC [35]. Recently, the role of activated STAT3 as the transcription factor induced by IL-10 in tolerogenic DC was investigated [36,37]. DCs expressing activated STAT3 produce less IL-12p40 by inhibiting the recruitment but not activation of NF-κB subunits [38]. In this study, we found that Shc blockade significantly enhances STAT3 phosphorylation on tyrosine residue-705 and increases the nuclear localization of phospho-STAT3 in a Src-dependent manner. This finding establishes a novel signaling event for STAT3 and associates its activation with a negative regulatory role for Shc in tolerogenic properties of DC. Furthermore, it has been found that the STAT3 pathway is required for IL-10-mediated down-regulation of inflammatory genes in monocytes by LPS [39,40], and STAT3 phosphorylation is likely due to LPS-induced IL-10 secretion in DC, while no phospho-STAT3 was observed in LPS-stimulated IL-10^-/- ^BMDCs [38]. Our results are consistent with these studies in that LPS-induced STAT3 phosphorylation was blocked by IL-10 neutralization. LPS-induced IκB degradation was not affected by Shc blockade and Src inhibitor SU6656 (data not shown). In conjunction with a similar activation profile for Erk1/2 and p38 signaling events, these data suggest that LPS-triggered Src-Shc signaling events are not involved directly in the signaling cascade which has been reported in causing increased IL-12p40 gene expression via other adaptor proteins through MAPK and NF-κB pathways [39,41]. The TLR-stimulated signaling cascades via activation of transcription factors NF-κB, AP-1 and IRF3, results in the expression of "innate" genes involved in pro-inflammatory properties of mature DC were not determined by Src-family kinase activity. However, the pro-inflammatory properties of mature DC were directed to Th2 phenotype and determined by Src-mediated STAT3 activation while Shc was blocked.

Furthermore, Shc proteins are key components of the pathways that activate downstream signaling molecules of growth factor receptors, extracellular matrix proteins and mechanical forces [16,17,42], and have been revealed a role in inflammatory signaling with increased ECM binding [43]. A recent study also showed that LPS-induced DC is enriched in MHC-II antigen and intercellular adhesion molecule 1 (ICAM-1) in the vesicular compartment called exosomes [44]. The enriched MHC-II and ICAM-1 are required for mature DC to prime naïve T cells representing the immunogenic activity of LPS-treated DCs. We briefly found that co-immunoprecipitation of p52Shc and ICAM-1 in LPS-treated DCs (data not shown) implying the involvement of adhesion molecules via Shc in modulating the immunogenic and tolerogenic properties of mature DC. However, the detailed mechanisms for the interplay of extracellular matrix, adaptor proteins as well as specific SFKs are critical in the regulation of DC-mediated immune response and require further investigation.

## Conclusions

In summary, we propose a model where the LPS-primed mature DC produce high level of IL-10 as well as nuclear transactivation of Src-mediated phospho-STAT3 while Shc-mediated signaling is blocked. Our findings suggest that the balance of inflammatory versus anti-inflammatory cytokines generated by DCs upon TLR stimulation could be critically determined by Shc, which may act like a switch in response to subsequently acquired immunity.

## Methods

### Rat

Four-week-old male Lewis (LEW; RT1^l^) rats were obtained from the National Laboratory Animal Breeding and Research Center (Taipei, Taiwan). All animals were maintained in specific pathogen-free animal facilities with water and commercial rat food provided *ad libitum*. Our experimental design was reviewed and approved by our institutional experimental animal committee.

### Preparation, transfection and treatment of bone marrow-derived dendritic cells

Thigh bones obtained from LEW rats were cleaned of muscle tissue and placed in sterile Petri dishes containing complete medium [RPMI-1640 with 10% fetal bovine serum (FBS), penicillin/streptomycin 100 U/ml and 100 mg/ml and 2 mM L-glutamine], following the procedures described elsewhere [45]. On day 8, the non-adherent cells were aspirated for DCs culture. Briefly, the wells were washed once with medium, and the cells were pooled. DCs (1 × 10^6 ^cells) were placed in a new six-well plate and cultured in fresh DC culture medium (DCM) containing 50 ng/ml recombinant granulocyte-monocyte colony-stimulating factor (rGM-CSF), 50 ng/ml rIL-4 (CytoLab, Ltd, Rehovot, Israel) and endotoxin (lipopolysaccharide, LPS; 0.5 μg/ml; Sigma, St Louis, MO, USA) with the addition of anti-Shc monoclonal antibody (mAb, 2 μg/ml) (Santa Cruz Biotechnology, Santa Cruz, CA, USA) and mouse IgG (2 μg/ml) (Santa Cruz Biotechnology) for 24 h. For transient transfection experiments, DCs were transfected with 50 nM of Shc siRNA or Silencer^® ^negative control siRNA (NEG. siRNA) (Ambion Inc., Austin, TX, USA) using GenMute™ (SignaGen Laboratories, Gaithersburg, MD, USA) for 24 h.

In some experiments, DCs (1 × 10^6 ^cells) were treated with a selective Src family kinase inhibitor SU6656 (10 nM) (Sigma, St. Louis, MO, USA) in the presence of LPS stimulation with or without Shc blockade.

### Cytokine detection in ELISA

After 24-h LPS stimulation of DCs with or without Shc blockade, supernatants were harvested and assayed for cytokine production using commercially available ELISA specifically for the rat IL-12p70 (eBioscience Inc., San Diego, CA, USA), IL-10 (R&D Systems Inc., Minneapolis, MN, USA) and IL-6 (R&D Systems Inc.) according to the manufacturer's instructions.

### RNA isolation and real-time PCR

Total RNA was isolated from DCs using the RNeasy kit from Qiagen (Valencia, CA, USA), according to the manufacturer's instructions. Reverse transcripts were performed using the First-Strand cDNA synthesis kit (Promega, Madison, WI, USA) following the manufacturer's recommendations. Total RNA (1 μg) was transcribed to cDNA in a 20-μl reaction volume. The quantitative PCR reaction was performed on an ABI 7500 Fast Real-Time PCR System with the SDS 1.4 program and using the ABI TaqMan Fast Universal PCR master mix (Applied Biosystems, Foster, CA, USA). The primers and TaqMan MGB probes of IL-10 (Rn00563409_m1) and IL-12 (Rn00575112_m1) were obtained from Applied Biosystems, and the final concentration of primers and probes was 300 nM and 250 nM, respectively. The cycling profile for each run was 95°C for 20 seconds and 40 cycles of 95°C for 3 seconds followed by 60°C for 30 seconds, using the default ramp rate. Normalization was performed by using rat GAPDH primers. Comparative real-time PCR (RT-PCR) data was analyzed in triplicate, including non-template controls. Fold increase in the expression of cytokine mRNA was calculated by using the comparative 2^ΔCt ^method.

### Antibodies and Western blot analysis

Cell lysates (30-40 μg) were electrophoresed and performed Western blot analysis as previously described [46]. Antibodies to phospho-Shc (Tyr239/240 or Tyr317) and total Shc, phospho- and total STAT3 were all purchased from Cell Signaling (Cell Signaling Technology, Inc., Danvers, MA, USA). Antibody to Shc blocking (PG-797) was purchased from Santa Cruz Biotechnology (Santa Cruz, CA, USA). Antibody to IL-10 neutralization (ARC9102) was purchased from Invitrogen (Carlsbad, CA, USA). Antibody to α-actin was obtained from Santa Cruz Biotechnology. Appropriate HRP-conjugated, secondary antibodies were used to visualize the specific bands. Detection was performed with the Immobilon Western HRP Substrate (Millipore, Billerica, MA, USA), and relevant bands were quantified by densitometry using UVI photo version 99 and TotalLab software version 1.00 (Nonlinear USA Inc., Durham, NC, USA).

### Nuclear and cytoplasmic protein extraction

DCs with different treatment conditions were washed twice in PBS, harvested, and pelleted by centrifugation at 500 × g for 3 min. CERI (NE-PER™, Thermo Fisher Scientific Inc., Rockford, IL, USA) was added to each sample following the supplier's guideline (200 μl of CERI per 20-μl cell volume). Briefly, cells were resuspended by vortexing for 15 seconds and then incubated on ice for 10 min. CERII was then added to each sample (11 μl of CERII solution for every 20-μl cell volume) followed by repeated vortexing and centrifugation at 13,000 × g for 5 min. The supernatant (cytoplasmic extract) was immediately stored on ice. The insoluble nuclear pellet was resuspended in 100 μl of NER (NE-PER™, Thermo Fisher Scientific Inc.) per 20-μl cell volume and incubated on ice for 40 min with intermittent brief vortexing. The samples were centrifuged at 13,000 × g for 10 min, and the supernatant (nuclear extract) was stored on ice. After measuring the total volumes of each fraction, their protein concentrations were measured using the Thermo protein assay reagent. All cytoplasmic and nuclear protein fractions were stored at -80°C and ready for further Western blot analysis.

### FACS analysis

DCs (1 × 10^6 ^cells/ml) were harvested in RPMI complete medium and were washed twice with PBS containing 0.1% sodium azide plus 2% heat-inactivated FBS (wash buffer). Cells were incubated with various mAb [fluorescein isothiocyanate (FITC)-anti-CD80, FITC-anti-CD86, FITC-anti-MHC class II] at 4°C for 40 min. All the fluorescein-conjugated mAb were obtained from BD Pharmingen (Franklin Lakes, NJ, USA) or eBioscience, and isotype-matched Ab used as controls were purchased from Santa Cruz Biotechnology. After extensive washing, the cells were fixed in 4% paraformaldehyde. Stained DC, gated according to forward- and side-scatter characteristics, were analyzed on Epics^® ^ALTRA™ flow cytometer (Beckman Coulter, Inc., Fullerton, CA, USA) using EXPO32 software. Fluorescence data were expressed as percentage of positive cells compared to immature DCs.

### Statistical analysis

The statistical significance of the data was calculated using Student's *t*-test. A value of *P *< 0.05 was considered to indicate statistical significance.

## List of abbreviations

APC: antigen presenting cell; BMDCs: bone marrow-derived dendritic cells; DCs: dendritic cells; ECM: 1/2; GAPDH: glyceraldehyde 3-phosphate dehydrogenase; HRP: horse radish peroxidase; IFNs: interferons; IL-extracellular matrix; ELISA: enzyme-linked immunosorbent assay; ERK 1/2: extracellular signal-regulated kinase 10: interleukin-10; IL-12: interleukin-12; IL-6: interleukin-6; LPS: lipopolysaccharide; mAb: monoclonal antibody; NF-κB: nuclear factor-kappa B; rGM-CSF: recombinant granulocyte-monocyte colony-stimulating factor; RT-PCR: real-time polymerase-chain reaction; rIL-4: recombinant interleukin-4; SFKs: Src-family kinases; Shc: Src homology 2 domain containing; siRNA: small interfering RNA; STAT3: signal transducer and activator of transcription 3; TLRs: toll-like receptors.

## Authors' contributions

KDC designed research, performed research, analyzed data and wrote the paper; LWH designed research, performed research; SG designed research and wrote the paper; CWY performed research and analyzed data; TN contributed analytical tools and platforms; CYL performed research; CHH performed research; YCC performed research; CCW contributed analytical tools and platforms; YFC contributed analytical tools and platforms KWC contributed analytical tools and platforms CCL contributed analytical tools and platforms CLC designed research and wrote the paper. All authors read and approved the final manuscript.

## References

[B1] Steinman RM Kaplan G Witmer MD Cohn ZA Identification of a novel cell type in peripheral lymphoid organs of mice. V. Purification of spleen dendritic cells, new surface markers, and maintenance *in vitro* J Exp Med 1979 149 1 16 10.1084/jem.149.1.1 762493 PMC2184752

[B2] Banchereau J Steinman RM Dendritic cells and the control of immunity Nature 1998 392 245 252 10.1038/32588 9521319

[B3] Lanzavecchia A Sallusto F Regulation of T cell immunity by dendritic cells Cell 2001 106 263 266 10.1016/S0092-8674(01)00455-X 11509174

[B4] Maldonado-Lopez R Moser M Dendritic cell subsets and the regulation of Th1/Th2 responses Semin Immunol 2001 13 275 282 10.1006/smim.2001.0323 11502162

[B5] Moser M Murphy KM Dendritic cell regulation of TH1-TH2 development Nat Immunol 2000 1 199 205 10973276 10.1038/79734

[B6] Chakraborty A Li L Chakraborty NG Mukherji B Stimulatory and inhibitory maturation of human macrophage-derived dendritic cells Pathobiol 1999 67 282 286 10.1159/000028080 10725803

[B7] Chakraborty A Li L Chakraborty NG Mukherji B Stimulatory and inhibitory maturation of human myeloid dendritic cells Clin Immunol 2000 94 88 98 10.1006/clim.1999.4826 10637093

[B8] Corinti S Albanesi C la Sala A Pastore S Girolomoni G Regulatory activity of autocrine IL-10 on dendritic cell functions J Immunol 2001 166 4312 4318 11254683 10.4049/jimmunol.166.7.4312

[B9] Willems F Marchant A Delville JP Gérard C Delvaux A Velu T de Boer M Goldman M Interleukin-10 inhibits B7 and intercellular adhesion molecule-1 expression on human monocytes Eur J Immunol 1994 24 1007 1009 10.1002/eji.1830240435 7512027

[B10] Allavena P Piemonti L Longoni D Bernasconi S Stoppacciaro A Ruco L Mantovani A IL-10 prevents the generation of dendritic cells from CD14+ blood monocytes, promotes the differentiation to mature macrophages and stimulates endocytosis of FITC-dextran Adv Exp Med Biol 1997 417 323 327 9286381 10.1007/978-1-4757-9966-8_53

[B11] De Smedt T Van Mechelen M De Becker G Urbain J Leo O Moser M Effect of interleukin-10 on dendritic cell maturation and function Eur J Immunol 1997 27 1229 1235 10.1002/eji.1830270526 9174615

[B12] Battaglia M Gregori S Bacchetta R Roncarolo MG Tr1 cells: from discovery to their clinical application Semin Immunol 2006 18 120 127 10.1016/j.smim.2006.01.007 16464609

[B13] Zhou X Schmidtke P Zepp F Meyer CU Boosting interleukin-10 production: therapeutic effects and mechanisms Curr Drug Targets Immune Endocr Metabol Disord 2005 5 465 575 10.2174/156800805774912926 16375698

[B14] Sallusto F Lanzavecchia A Efficient presentation of soluble antigen by cultured human dendritic cells is maintained by granulocyte/macrophage colony-stimulating factor plus interleukin 4 and downregulated by tumor necrosis factor alpha J Exp Med 1994 179 1109 1118 10.1084/jem.179.4.1109 8145033 PMC2191432

[B15] Stefanova I Corcoran ML Horak EM Wahl LM Bolen JB Horak ID Lipopolysaccharide induces activation of CD14-associated protein tyrosine kinase p53/56lyn J Biol Chem 1993 268 20725 20728 7691802

[B16] Ravichandran KS Signaling via Shc family adapter proteins Oncogene 2001 20 6322 6330 10.1038/sj.onc.1204776 11607835

[B17] Chen KD Li YS Kim M Li S Yuan S Chien S Shyy JY Mechanotransduction in response to shear stress. Roles of receptor tyrosine kinases, integrins, and Shc J Biol Chem 1999 274 18393 18400 10.1074/jbc.274.26.18393 10373445

[B18] Kavanaugh WM Williams LT An alternative to SH2 domains for binding tyrosine-phosphorylated proteins Science 1994 266 1862 1865 10.1126/science.7527937 7527937

[B19] Finetti F Pellegrini M Ulivieri C Savino MT Paccagnini E Ginanneschi C Lanfrancone L Pelicci PG Baldari CT The proapoptotic and antimitogenic protein p66SHC acts as a negative regulator of lymphocyte activation and autoimmunity Blood 2008 111 5017 5027 10.1182/blood-2007-12-130856 18334675

[B20] Pulendran B Kumar P Cutler CW Mohamadzadeh M Van Dyke T Banchereau J Lipopolysaccharides from distinct pathogens induce different classes of immune responses *in vivo* J Immunol 2001 167 5067 5076 11673516 10.4049/jimmunol.167.9.5067 PMC3739327

[B21] Pulendran B Variegation of the immune response with dendritic cells and pathogen recognition receptors J Immunol 2005 174 2457 2465 15728447 10.4049/jimmunol.174.5.2457

[B22] Poltorak A He X Smirnova I Liu MY Van Huffel C Du X Birdwell D Alejos E Silva M Galanos C Freudenberg M Ricciardi-Castagnoli P Layton B Beutler B Defective LPS signaling in C3H/HeJ and C57BL/10ScCr mice: mutations in Tlr4 gene Science 1998 282 2085 2088 9851930 10.1126/science.282.5396.2085

[B23] Means TK Golenbock DT Fenton MJ The biology of Toll-like receptors Cytokine Growth Factor Rev 2000 11 219 232 10.1016/S1359-6101(00)00006-X 10817965

[B24] Underhill DM Ozinsky A Hajjar AM Stevens A Wilson CB Bassetti M Aderem A The Toll-like receptor 2 is recruited to macrophage phagosomes and discriminates between pathogens Nature 1999 401 811 815 10.1038/44605 10548109

[B25] Brightbill HD Libraty DH Krutzik SR Yang RB Belisle JT Bleharski JR Maitland M Norgard MV Plevy SE Smale ST Brennan PJ Bloom BR Godowski PJ Modlin RL Host defense mechanisms triggered by microbial lipoproteins through toll-like receptors Science 1999 285 732 736 10.1126/science.285.5428.732 10426995

[B26] Means TK Wang S Lien E Yoshimura A Golenbock DT Fenton MJ Human toll-like receptors mediate cellular activation by Mycobacterium tuberculosis J Immunol 1999 163 3920 3927 10490993

[B27] Kapsenberg ML Dendritic-cell control of pathogen-driven T-cell polarization Nat Rev Immunol 2003 3 984 993 10.1038/nri1246 14647480

[B28] Janssen E Zhang W Adaptor proteins in lymphocyte activation Curr Opin Immunol 2003 15 269 276 10.1016/S0952-7915(03)00044-X 12787751

[B29] Lee MS Kim YJ Signaling pathways downstream of pattern-recognition receptors and their cross talk Annu Rev Biochem 2007 76 447 480 10.1146/annurev.biochem.76.060605.122847 17328678

[B30] Li X Qin J Modulation of Toll-interleukin-1 receptor mediated signaling J Mol Med 2005 83 258 266 10.1007/s00109-004-0622-4 15662540

[B31] Pacini S Pellegrini M Migliaccio E Patrussi L Ulivieri C Ventura A Carraro F Naldini A Lanfrancone L Pelicci P Baldari CT p66SHC promotes apoptosis and antagonizes mitogenic signaling in T cells Mol Cell Biol 2004 24 1747 1757 10.1128/MCB.24.4.1747-1757.2004 14749389 PMC344195

[B32] Salcini AE McGlade J Pelicci G Nicoletti I Pawson T Pelicci PG Formation of Shc-Grb2 complexes is necessary to induce neoplastic transformation by overexpression of Shc proteins Oncogene 1994 9 2827 2836 8084588

[B33] Gotoh N Tojo A Shibuya M A novel pathway from phosphorylation of tyrosine residues 239/240 of Shc, contributing to suppress apoptosis by IL-3 EMBO J 1996 15 6197 6204 8947042 PMC452441

[B34] Patrussi L Savino MT Pellegrini M Paccani SR Migliaccio E Plyte S Lanfrancone L Pelicci PG Baldari CT Cooreration and selectivity of the two Grb2 binding sites of p52Shc in T-cell antigen receptor signaling to ras family GTPases and Myc-dependent survival Oncogene 2005 24 2218 2228 10.1038/sj.onc.1208384 15688026

[B35] Wan H Versnel MA Leijten LM van Helden-Meeuwsen CG Fekkes D Leenen PJ Khan NA Benner R Kiekens RC Chorionic gonadotropin induces dendritic cells to express a tolerogenic phenotype J Leukoc Biol 2008 83 894 901 10.1189/jlb.0407258 18171698

[B36] Murray P The primary mechanism of the IL-10-regulated anti-inflammatory response is to selectively inhibit transcription Proc Natl Acad Sci USA 2005 102 8686 8691 10.1073/pnas.0500419102 15937121 PMC1150817

[B37] Murray P Understanding and exploiting the endogenous interleukin-10/STAT3-mediated anti-inflammatory response Curr Opin Pharmacol 2006 6 379 386 10.1016/j.coph.2006.01.010 16713356

[B38] Hoentjen F Sartor RB Ozaki M Jobin C STAT3 regulates NF-kappaB recruitment to the IL-12p40 promoter in dendritic cells Blood 2005 105 689 696 10.1182/blood-2004-04-1309 15251981

[B39] Takeda K Akira S Toll-like receptors in innate immunity Int Immunol 2005 17 1 14 15585605 10.1093/intimm/dxh186

[B40] Riley JK Takeda K Akira S Schreiber RD Interleukin-10 receptor signaling through the JAK-STAT pathway. Requirement for two distinct receptor-derived signals for anti-inflammatory action J Biol Chem 1999 274 16513 16521 10.1074/jbc.274.23.16513 10347215

[B41] Land WG Innate immunity-mediated allograft rejection and strategies to prevent it Transplant Proc 2007 39 667 672 10.1016/j.transproceed.2007.01.052 17445569

[B42] Pelicci G Lanfrancone L Grignani F McGlade J Cavallo F Forni G Nicoletti I Grignani F Pawson T Pelicci PG A novel transforming protein (SHC) with an SH2 domain is implicated in mitogenic signal transduction Cell 1992 70 1165 1174 10.1016/0092-8674(92)90536-l 1623525

[B43] Liu Y Sweet DT Irani-Tehrani M Maeda N Tzima E Shc coordinates signals from intercellular junctions and integrins to regulate flow-induced inflammation J Cell Biol 2008 182 185 196 10.1083/jcb.200709176 18606845 PMC2447891

[B44] Segura E Nicco C Lombard B Veron P Raposo G Batteux F Amigorena S Thery C ICAM-1 on exosomes from mature dendritic cells is critical for efficient naïve T-cell priming Blood 2005 106 216 223 10.1182/blood-2005-01-0220 15790784

[B45] Hsu LW Goto S Nakano T Lai CY Kao YH Lin YC Kawamoto S Ono K Lord R Goto T Omori N Sato S Chiang KC Chen SH Jawan B Cheng YF Chiu KW Chen CL The effects of anti-histone H1 antibody on immune cells responsible for rejection reaction Mol Immunol 2005 42 1155 1164 10.1016/j.molimm.2004.11.010 15829305

[B46] Hsu LW Chen CL Nakano T Lai CY Chiang KC Lin YC Kao YH Chen SH Goto T Sung WC Yang CH Cheng YF Jawan B Chiu KW Goto S The role of a nuclear protein, histone H1, on signaling pathways for the maturation of dendritic cells Clin Exp Immunol 2008 152 576 584 10.1111/j.1365-2249.2008.03652.x 18435805 PMC2453206

